# “Normal” vs. “difficult” cases with eating disorders: the therapists’ perspective

**DOI:** 10.3389/fpsyg.2026.1761349

**Published:** 2026-02-18

**Authors:** Armin Hartmann, Inga Lau, Carolin Klose, Milena Egenolf, Almut Zeeck

**Affiliations:** Department of Psychosomatic Medicine und Psychotherapy, Center for Mental Health, Faculty of Medicine, University of Freiburg, Freiburg, Germany

**Keywords:** case difficulty, eating disorder, psychotherapy, severity, therapist perspective

## Abstract

**Introduction:**

Patients with an eating disorder (ED) are considered difficult to treat. We aimed to identify the characteristics of patients with an ED, which are considered especially challenging to treat from the therapist’s perspective.

**Methods:**

Psychotherapists with experience in ED treatment were asked to describe a “normal” as well as a “difficult” case they treated lately, using an online-survey. They described their patients by filling in a list of symptoms and their severity, on personality traits and functioning as well as a list of possible therapeutic challenges experienced over the course of treatment.

**Results:**

127 psychotherapists were recruited. “Difficult” cases were characterized by variables which could be related to three areas: patients´ motivation (higher level of ambivalence), interactional behavior/personality traits (more antagonism) and somatic complications, such as electrolyte imbalance. Ambivalence was the strongest predictor of case difficulty, followed by patient personality. Therapist or work place characteristics were not associated with experiencing treatments as “normal” or “difficult.”

**Discussion:**

In particular, aspects related to the therapeutic process and relationship characterize the group of “difficult” cases from a therapist’s view.

## 1 Introduction

Eating disorders (EDs) are considered difficult to treat (Thompson-Brenner et al., 2012). Challenges in the treatment of EDs may include the limited treatment success (only about half of adult patients with anorexia nervosa (AN) or bulimia nervosa (BN) recover), the self-damaging character of ED symptoms, somatic complications or comorbid conditions such as an additional personality disorder or posttraumatic stress disorder (Abbate-Daga et al., 2013; Colli et al., 2015; Davén et al., 2022). Overall, one might assume that the most challenging treatments are those with patients of higher symptom severity.

The classification systems DSM-5 and ICD-11 in their most recent version entail criteria for “severity” of AN and BN. The DSM-5 severity classification for patients with AN is oriented on the body mass index (BMI) and defines four levels (mild: BMI ≥ 17kg/m^2^; moderate: 16–16.99 kg/m^2^; severe: 15–15.99 kg/m^2^; extreme: <15 kg/m^2^) (American Psychiatric Association, 2013), while the ICD-11 distinguishes two groups: patients with a *significantly low* body weight (BMI ≥ 14 kg/m^2^) and those with a *dangerously low* body weight (BMI < 14 kg/m^2^) (American Psychiatric Association, 2013; de Zwaan, 2025). Although several studies found that a lower BMI is associated with poorer outcomes [e.g., (Herzog et al., 2022)], the four DSM-5 categories seem to have limited predictive power to detect differences in treatment outcome and recovery (Dang et al., 2025). Furthermore, the DSM-5 severity criteria were considered not very helpful by clinicians (e.g., lacking clinical relevance) (Dang et al., 2025). In terms of ICD-11 categories, there is still a paucity of studies showing their clinical usefulness (Dang et al., 2025). For BN, only the DSM-5 defines severity of symptomatology, oriented on the frequency of compensatory behavior (mild: 1–3 episodes/week; moderate: 4–7 episodes/week; severe: 8–13 episodes/week; extreme: 14 or more episodes/week) (American Psychiatric Association, 2013). In contrast to AN, the severity criteria for BN were found to be related to outcome and considered helpful (Dang et al., 2025).

But what is a challenging and difficult case from the therapist’s point of view? Are these cases the ones with more severe underweight and more inappropriate compensatory behaviors?

Several studies point to the fact that patients with an ED in general are a challenging group to treat (Zeeck et al., 2025). In particular, they can induce a range of intense and often negative emotional reactions in therapists (Colli et al., 2015; Satir et al., 2009). Negative emotions include helplessness, frustration, anger or fear. These emotional reactions may be considered an indicator of treatment difficulties. They could be triggered by ED symptoms such as severe underweight, but also by difficulties which emerge during the treatment process including problems in building a trustful therapeutic relationship or patients´ rejection of help (Zeeck et al., 2025).

In this study, which is part of a larger study on therapist’s emotional reactions to patients with an ED, we aimed to identify patients’ characteristics which discriminate “difficult” psychotherapy cases from “normal” cases *from the perspective of the therapist.* We distinguished three groups of patient variables: (1) the symptom spectrum of a case (including the severity of a symptom), (2) personality functioning and personality traits and (3) “therapeutic challenges” which may occur during treatment. Symptoms and personality are typically assessed at the beginning of a treatment, while therapeutic challenges will emerge during the course of psychotherapy. The challenges may originate from the eating disorder itself (e.g., patients ambivalence concerning change, stagnation of weight gain or weight loss), from personality pathology (e.g., self-harm or suicidality, interactional behavior), from traumatization (e.g., frequent dissociation), or from external sources (e.g., parents, social, or legal problems).

The presented study is exclusively investigating the view of therapists, with no other source of information. While some of the variables collected appear to be “objective” (e.g., BMI, personality), we only collect data on the therapists’ inner worlds. We assume that therapist characteristics will not strongly determine the reporting of presenting ED symptoms or the personality of a patient, but the perceived challenges over the course of therapy could be biased by the therapist’s theoretical orientation, experience, personality or attachment style. Therefore, we took care to describe the therapist sample in great detail and we investigated the relations between therapist’s characteristics, workplace variables and their case descriptions.

We assumed the following: If case severity is the main source of difficulty, variables of the first two groups (symptoms and personality) must emerge as significant predictors and the largest amount of explained variance must be attributable to them. If the unfolding of challenges in therapy is the main source of difficulty, variables of the third group must outperform the predictions from ED symptoms and personality pathology.

Our hypotheses were:

(A) All three groups of predictors will distinguish the “difficult” case from the “normal” case, but ED and personality symptoms will be outperformed by the predictive power (*r*^2^) of encountered therapeutic challenges;
(B) Therapist characteristics will only marginally bias the reports of presented symptoms (less than 5% of explained variance) or therapeutic challenges.

## 2 Materials and methods

### 2.1 Study design

German speaking psychotherapists were asked to describe a “normal” case and a “difficult” case of a patient with an ED treated recently. Data were retrieved by an online-survey. The aim was to recruit a minimum of 100 therapists from different theoretical orientations and treatment settings (private practice, hospital). The study was approved by the local ethics committee (No 24-1297) and supported by the Heidehof-Stiftung Stuttgart (Project No 59055.05.1/1-24).

### 2.2 Participants

Therapists were recruited through the German Eating Disorder Society (DGESS), the Austrian Society for Eating Disorders, regional eating disorder networks, through hospitals specialized in treating patients with EDs and counselling centers. Recruitment took place between Oct 21st 2024 and May 6th 2025.

### 2.3 Inclusion criteria

We included licensed psychotherapists or advanced trainees in the therapy methods which are reimbursed by the German health care system (cognitive-behavioral therapy [CBT]; psychodynamic therapy incl. psychoanalysis [PD]; systemic therapy [ST]). We included therapists for adults as well as for children and adolescents. In Germany licensed psychotherapists for adults have a professional background as psychologists or physicians; licensed psychotherapists for children and adolescents may also be social workers or social pedagogues with training in one of the above-mentioned treatment modalities.

### 2.4 Procedure

Therapists were asked to describe two recent therapy cases with the main diagnosis of an ED, one experienced as a “normal” case, the other as a “difficult” and challenging case. The diagnosed ED should qualify for an ICD-10 diagnosis, including anorexia nervosa, bulimia nervosa, binge eating disorder, atypical EDs or EDs not otherwise specified. The difficult case was defined as “difficult,” “hard to treat”, or “troublesome”, as compared to the “normal” case defined as “normal difficulty” and “uncomplicated.”

The descriptions of the cases represent the therapist’s view of the case. Patients were never given any measures for self-rating and were never approached by the research team. Self-report questionnaires belonging to the case description (e.g., PID5BF, OPD-SQ) were answered by the therapists to their best understanding of their patients.

Therapists received 75€ for a complete data set and feed-back forms on individual scores from the standardized instruments of the survey.

### 2.5 Measures

#### 2.5.1 Therapists

Therapists were asked for their age and gender as well as training in psychotherapy. They further characterized their therapeutic orientation by rating the influence of therapy theories in their daily practice on a rating scale ranging from 0 to 5 (not at all to very much) using the list of theoretical orientations as provided by Orlinsky and Ronnestad (2005), extended by 3rd wave CBT therapy “clusters” (see Table 1). We further asked if therapists had completed their formal training with an approbation and if they were aiming for or had acquired approbations for adult or child & youth therapy or both, since in Germany each requires a distinct training pathway and examination. Different from the “official” qualification, therapist often use techniques and methods form other therapy schools (Orlinsky and Ronnestad, 2005). Therefore, we also asked them how much their therapeutic approach was influenced by other, broadly available therapy trainings or schools. The provided spectrum included on the PD side also Mentalization Based Treatment (MBT) and Transference Focused Therapy (TFP); on the CBT side, behavioral and cognitive orientations were differentiated and three 3rd wave facets (CBASP/Schema Therapy; Mindfulness/Acceptance/Emotion Focussed Therapy; DBT) could be rated. Further, Systemic Therapy, Humanistic (Rogerian) Therapy; Interpersonal Therapy, and integrative approaches could be chosen.

**Table 1:** Psychotherapist sample.

Variable	Scale/values	M (SD); *N* (%)
Age	Years	36.3 (11.0)
Gender	Female Male Non-binary	104 (81.9%) 23 (18.1%) 0 (0.0%)
Living situation	Married In a relationship Single *N* missing	53 (41.7%) 54 (42.5%) 16 (12.6%) 4 (3.2%)
Qualification	Psychology (MSc, Diploma) Medicine Psychology & psychotherapy (MSc) Social work or paedagogics Psychotherapy (MSc) Psychology & medicine Other combination	86 (67.7%) 25 (19.7%) 5 (3.9%) 5 (3.9%) 2 (1.6%) 2 (1.6%) 2 (1.6%)
Approbation	Yes	68 (54.0%)
Licensed for…	Cognitive behavioral (CBT) Psychodynamic therapy/psychoanalysis Systemic therapy Psychodynamic & systemic therapy CBT & psychodynamic therapy	80 (63.0%) 36 (28.3%) 5 (3.9%) 4 (3.1%) 2 (1.6%)
License for…	Adults Adults & child/youth Child/youth	95 (74.8%) 14 (1.0%) 18 (14.2%)
Influenced by treatment orientation…	0–5 (not at all → very much) Psychoanalysis/psychodynamic Mentalization based therapy (MBT) Transference focused therapy (TFP) BT behavioral therapy CBT cognitive behavioral CBT/3rd wave CBASB/schema CBT/3rd wave (mindfulness/acceptance/emotion) CBT/3rd wave DBT Systemic/family therapy Humanistic (e.g., rogerian) Interpersonal therapy Integrative therapy Other	2.2 (2.0) 1.6 (1.5) 0.6 (1.1) 3.3 (1.6) 3.5 (1.6) 2.2 (1.6) 2.6 (1.6) 2.3 (1.6) 2.1 (1.4) 1.5 (1.5) 1.5 (1.5) 1.6 (1.7) 0.6 (1.3)
Personality traits PID5BF+ z-Scores	Negative affectivity Detachment Antagonism Disinhibition Psychoticism Anankastia	−0.6 (0.7) −0.5 (0.6) −0.3 (0.9) −0.7 (0.9) −0.6 (0.6) −0.3 (0.9)
Attachment ECR-R8	Attachment anxiety Avoidant attachment	2.0 (1.1) 1.9 (0.8)
Main workplace (≥ 50% of weekly working hours)	Inpatient unit/day hospital Private practice Other (social institutions, university, etc.)	87 (68.5%) 15 (11.8%) 25 (19.7%)
Number of sessions	Per week	12.5 (7.2)
Treatment setting	Adult (individual therapy) Child/Youth (individual therapy) Couple therapy Family therapy Groups Other	8.7 (10.9) 1.9 (3.9) 0.3 (1.2) 0.5 (1.9) 2.1 (3.5) 0.7 (3.0)
Control over working conditions	0–5 (no control – full control)	2.5 (1.2)
ED supervision available	Yes	87 (86.5%)
Satisfaction with ED-supervision	0–5 (not at all – very much satisfied)	4.4 (0.6)

##### 2.5.1.1 Workplace

Therapists specified the number of hours per week they worked in a hospital/day hospital, private practice, or other institutions. They indicated whether eating disorder-specific supervision was available and, if so, how satisfied they were with it.

##### 2.5.1.2 Personality

Therapists filled in measures on personality traits (PID5BF+, see below). It is important to note, that in the therapist sample we did not aim to measure relevant levels of maladaptive personality traits or functioning, but dimensional accentuations of a therapist’s personality.

##### 2.5.1.3 Attachment style

Attachment style was assessed with the ECR-RD8, a brief self-report measure of adult attachment that assesses two key dimensions: attachment-related anxiety and attachment-related avoidance. It was derived from the full Experiences in Close Relationships–Revised (ECR-R) questionnaire (Fraley et al., 2000). Ehrenthal et al. (2021) developed the eight-item version (ECR-RD8) using datasets from multiple studies. The ECR-RD8 retains the original two-factor structure with good reliability (McDonald’s *ω* = 0.83 for anxiety; *ω* = 0.82 for avoidance).

#### 2.5.2 Patients

The most basic information was the patients’ age, gender and ICD-10 diagnosis of the eating disorder (see Table 2).

**Table 2:** Patient sample by difficulty.

Variable	Scale/values	Normal	Difficult	Δ
		M (SD); *N* (%)	M (SD); *N* (%)	
Age	Years	24.4 (9.6)	25.8 (10.4)	N.S.
Gender	F M Non-binary	122 (96.1%) 4 (3.2%) 1 (0.8%)	120 (94.5%) 6 (4.7%) 1 (0.8%)	N.S.
ICD-10 ED Diagnoses	AN, restrictive F50.0/0.00 AN, binge-purge F50.01 Atypical AN F50.1 BN F50.2 Atypical BN F50.3 EDNOS F50.4/0.8/0.9	55 (43.3%) 22 (17.3%) 21 (16.4%) 16 (12.6%) 1 (1.3%) 12 (9.5%)	48 (37.8%) 42 (33.1%) 19 (15.0%) 13 (10.2%) 0 (0.0%) 5 (3.9%)	N.S.
No. of comorbid psych. ICDs	Max 3	1.14 (0.85)	1.58 (0.90)	***
Most frequent comorbid psych. Diagnoses	F32 (MDE first episode) F33 (MDE recurrent) F43 (PTSD) F60 (personality disorder)	38 46 6 11	23 69 17 26	N.A.*
No. of comorbid somatic diagnoses	Max 2	0.45 (0.60)	0.80 (0.72)	***
BMIs	Lifetime MIN Lifetime max Therapy min Therapy max	16.0 (3.7) 21.8 (5.9) 18.0 (6.7) 21.0 (6.5)	14.9 (4.7) 21.1 (5.6) 16 (5.3) 18.6 (5.1)	* n.s. ** ***

The maximum number of comorbid diagnoses was limited by the number of presented fields in the survey (psych. =3, somatic = 2). *Frequencies are based on multiple responses. Δ = *t*-test for paired observations *; **; ***p < 0.05/0.01/0.001.

##### 2.5.2.1 Symptom spectrum: ED symptoms and general psychopathology

We compiled a list of symptoms describing EDs based on diagnostic criteria, ED literature and discussions in a group of ED experts. Weight pathology was measured with the lowest BMI in treatment. All other ED symptoms were rated on a scale from 0 (does not apply) to 4 (extreme). Further pathology considered comorbid psychiatric symptoms of affective and personality disorders (e.g., depression, anxiety, borderline PD). For a full list of presented symptoms see Table 3.

**Table 3:** Spectrum of symptoms.

Symptoms					Rotated factor pattern					
	Difficult (*N* = 124)	Normal (*N* = 118)		All cases (*N* = 242)	Psych.Sy.	Som.Sy.	Abuse	Bul	BMI+	Som/OCD
	M (SD)	M (SD)	Δ	M (SD)	SF1	SF2	SF3	SF4	SF5	SF6
BMI_treatMIN^pred^	15.50 (3.48)	16.63 (3.37)	**	16.06 (3.47)	−0.02	−0.26	0.06	0.14	**0.75**	−0.10
Exercising	2.28 (1.42)	2.11 (1.25)	N.S.	2.20 (1.34)	0.03	−0.02	0.18	−0.06	**−0.33**	0.01
Binges	0.84 (1.26)	0.97 (1.26)	N.S.	0.90 (1.26)	0.05	0.07	−0.03	**0.68**	**0.40**	−0.16
Purges^pred^	1.06 (1.49)	0.77 (1.27)	N.S.	0.92 (1.39)	0.11	0.10	0.10	**0.98**	0.04	−0.01
Lax.Abuse	0.51 (0.98)	0.19 (0.54)	*	0.35 (0.81)	0.05	0.18	**0.72**	0.06	−0.06	0.12
Diur.Abuse	0.20 (0.70)	0.10 (0.40)	N.S.	0.15 (0.57)	0.02	0.13	**0.85**	0.00	−0.05	0.12
Chewing/spitting	0.34 (0.83)	0.12 (0.42)	**	0.23 (0.67)	0.25	0.23	**0.40**	0.08	−0.11	−0.05
Regurgitation	0.16 (0.50)	0.08 (0.36)	N.S.	0.12 (0.44)	0.16	0.26	0.09	−0.07	−0.01	−0.13
Life threat weight	1.53 (1.51)	1.08 (1.31)	**	1.31 (1.43)	0.06	**0.45**	0.01	−0.11	−0.66	0.15
Electrolyte imbalance^pred^	1.20 (1.29)	0.50 (0.80)	***	0.86 (1.14)	0.14	**0.72**	0.11	0.11	−0.29	0.08
Dental damage	0.71 (1.12)	0.34 (0.73)	**	0.53 (0.97)	0.05	**0.59**	0.21	**0.34**	0.02	0.10
Osteoporosis	0.79 (1.16)	0.35 (0.77)	***	0.57 (1.01)	−0.02	**0.49**	0.20	0.03	−0.37	0.26
Organ lesions	0.60 (1.02)	0.13 (0.38)	***	0.37 (0.81)	0.14	**0.68**	0.14	0.02	−0.13	0.25
Traumatization	1.35 (1.38)	0.75 (1.21)	**	1.06 (1.33)	**0.51**	0.12	−0.06	−0.01	0.03	0.22
Self-harm	1.29 (1.34)	0.66 (1.01)	***	0.98 (1.23)	**0.73**	0.05	0.05	0.05	−0.12	−0.02
Emotional instability	2.00 (1.32)	1.07 (1.06)	***	1.55 (1.29)	**0.67**	0.05	0.09	0.11	0.02	0.15
Suicidality^pred^	1.41 (1.26)	0.66 (0.91)	***	1.05 (1.16)	**0.74**	0.05	0.11	0.03	−0.03	0.09
Depression	2.51 (0.97)	2.03 (1.02)	**	2.28 (1.02)	**0.60**	0.16	0.04	0.03	0.03	0.08
Anxiety	1.98 (1.28)	1.64 (1.14)	*	1.81 (1.22)	**0.42**	−0.02	0.06	−0.06	−0.14	**0.39**
OCD	1.43 (1.42)	0.69 (1.02)	***	1.07 (1.29)	0.18	0.23	0.05	−0.11	−0.21	**0.53**
Somatisation^pred^	1.16 (1.18)	0.53 (0.86)	***	0.86 (1.08)	0.26	0.11	0.16	−0.01	−0.03	**0.65**

Rating scale 0 to 4 (0 does not apply, 1 slightly, 2 marked, 3 strong, 4 extreme); Δ = *t*-test for paired observations *; **; ***p < 0.05/0.01/0.001; ^pred^selected as predictor for logistic regression; *Varimax rotated factor scores with EV >1, factor loadings >0.30 are bolded.

##### 2.5.2.2 Personality functioning and personality traits

Patient personality functioning was assessed using the OPD-Structure Questionnaire - short version (OPD-SQS). The OPD-SQS is a self-report questionnaire of 12 items (rating 0–4; “not at all” to “very much”) aiming to measure the overall level of personality functioning (Ehrenthal et al., 2015). It provides a single factor score (Cronbach’s *α* = 0.88). It was validated in a large representative German sample (Ehrenthal et al., 2023). Patient’s personality traits were assessed through the PID5BF + (Personality Inventory for DSM-5 and ICD-11 – Brief Form Plus). The PID5BF + is a self-report questionnaire designed to assess maladaptive personality traits according to the dimensional models of the DSM-5 (Alternative Model for Personality Disorders, AMPD, Criterion B) and ICD-11. It covers six trait-based domains: (1) Negative Affectivity, (2) Detachment, (3) Antagonism, (4) Disinhibition, (5) Psychoticism, and (6) Anankastia - (1) to (5) covering the DSM-V model, (1) to (4) & (6) covering the ICD-11 (see Table 4). A trait comprises three facets (e.g., antagonism: manipulativeness, deceitfulness & grandiosity). Each facet is measured by two items, rated on a Likert Scale (from 0 = “not at all” to 3 “completely true”). The reliability of the trait domains (mean *ω* = 0.77) and facets (mean *ω* = 0.80) is good. Normative data is available (Kerber et al., 2022). Convergent validity with other measures of the DSM-S alternative model has been established (Pires et al., 2023). It should be emphasized that we used these instruments as external assessments. The therapists answered the items as they assumed the patients would have done. In this respect, these scales measure the therapists’ perception of the patients, and there is no reliable measurement of the patients.

**Table 4:** Personality functioning and traits.

Personality	Difficult (*N* = 124)	Normal (*N* = 118)		All cases (*N* = 242)	Super-factors	
Functioning	M (SD)	M (SD)	Δ	M (SD)	PF1	PF2
OPD-SQS total ^pred^	2.57 (0.69)	1.89 (0.76)	***	2.24 (0.80)	**0.39**	**0.74**
PID5BF + z detachment^pred^	1.54 (1.15)	0.69 (1.11)	***	1.12 (1.21)	0.09	**0.73**
PID5BF + z anankastia	1.18 (1.08)	0.96 (1.39)	*	1.07 (1.24)	0.08	**0.30**
PID5BF + z disinhibition	0.19 (1.16)	−0.49 (1.24)	***	−0.14 (1.25)	**0.86**	0.13
PID5BF + z antagonism^pred^	1.43 (1.59)	0.11 (1.40)	***	0.79 (1.64)	**0.72**	0.13
PID5BF + z negative affect	0.74 (0.89)	0.50 (0.97)	*	0.62 (0.93)	**0.51**	0.18
PID5BF + z psychoticism	0.24 (0.97)	−0.46 (0.85)	***	−0.10 (0.97)	**0.66**	**0.38**

PID5BF + as z-scores, OPD-SQS-Total as raw score; Δ = *t*-test for paired observations *; **; ***p < 0.05/0.01/0.001; ^pred^selected as predictor for logistic regression; *Varimax rotated factor scores with EV >1; factor loadings >0.30 are bolded.

##### 2.5.2.3 Therapeutic challenges

We set up a list of therapeutic challenges which are typically encountered in the treatment of EDs [based Zeeck et al. (2025) and discussions in a group of ED experts]: For example, patients suffering from anorexia nervosa may show interruptions in weight gain and even weight reduction. When a therapy does not overcome this unwanted event and a patient does not gain weight at all or even worse, when she loses weight, then this is a strong challenge for therapy planning and process. All challenges are more or less likely, given the diagnoses and the history of the ED. If a patient has a Borderline PD diagnosis at intake, self-harm and emotional instability are likely to occur, but only the process of the treatment will show whether, and to what extent such symptoms occur. The full list of challenges is provided by Table 5. For both the “normal” and the “difficult” case the challenges were rated on a scale from 0 (not at all) to 4 (extreme).

**Table 5:** Therapeutic challenges.

Challenge					Super-factors		
	Difficult (*N* = 124)	Normal (*N* = 118)		All cases (*N* = 242)	Weight	Binge/purge	Trauma
	M (SD)	M (SD)	Δ	M (SD)	CF1	CF2	CF3
Ambivalence^pred^	3.29 (0.72)	1.87 (1.01)	***	2.60 (1.12)	**0.66**	0.07	0.18
Weight reduction	1.43 (1.22)	0.79 (0.98)	***	1.12 (1.15)	**0.50**	0.08	0.10
Deceiving behavior	2.05 (1.26)	0.73 (0.90)	***	1.40 (1.28)	**0.82**	0.02	0.18
Pressure from ext. sources	1.62 (1.39)	0.92 (1.03)	***	1.28 (1.27)	**0.44**	−0.05	0.27
More bingeing	0.59 (1.04)	0.34 (0.71)	*	0.47 (0.90)	−0.02	**0.67**	0.10
More purging^pred^	0.63 (1.05)	0.25 (0.64)	***	0.45 (0.89)	0.15	**0.99**	0.08
Complications of trauma^pred^	0.95 (1.25)	0.42 (0.90)	***	0.69 (1.13)	0.02	0.01	**0.54**
Social problems	1.89 (1.37)	1.00 (1.05)	***	1.45 (1.30)	**0.32**	0.09	**0.51**
More self-harm	0.96 (1.23)	0.33 (0.77)		0.65 (1.08)	0.25	0.20	**0.47**
Psychotic decompensation	0.34 (0.81)	0.06 (0.27)	***	0.20 (0.62)	0.20	0.06	**0.29**

Δ = *t*-test for paired observations *; **; ***p < 0.05/0.01/0.001; ^pred^selected as predictor for logistic regression; *Varimax rotated factor scores with EV >1; challenges sorted by [factor, loading]; WT = weight; CF1 = therapeutic challenges factor 1 Weight loss; CF2 = binge/purging behavior, CF3 = trauma related symptoms; factor loadings >0.30 are bolded.

### 2.6 Statistical analyses

Means, SDs, frequencies, and percentages were used to describe all variables. In the case descriptions, the variables are organized into three distinct categories: Spectrum of symptoms (ED and further symptoms related to comorbidity), personality (personality functioning, personality traits), and therapeutic challenges under treatment. For each group we computed a factor analysis (principal components, eigenvalue >1, varimax rotation). The respective rotated factor pattern is displayed in each category’s table of descriptive statistics. This procedure shows the pattern of covariances within each group of variables and reduces the complexity of the case description. It also guided the selection of predictor variables for regression models. Selecting only the highest loading variable from each orthogonal factor largely avoids multi collinearity in predictor sets of regression analyses.

Testing the differences of D/N case descriptions we apply t-tests for paired observations, since the case descriptions are nested in therapists. To examine whether the therapist characteristics are biasing their case descriptions we correlated their personality dimension scores and age with the case description variables. Multiple testing was controlled by a group wise (by symptoms, challenges, patient personality) Bonferroni correction.

We compared four logistic regression models. The difficulty of a case (dichotomous dependent variable = Difficult vs. Normal) is predicted by (1) the spectrum of symptoms, (2) personality functioning, (3) therapeutic challenges and (4) a combined set of all significant variables from steps (1) to (3).

All statistical analyses were computed with SAS-JMP V13.1 (SAS Institute Inc., 2017), (RRID: SCR_022199).

## 3 Results

### 3.1 Participants

127 therapists filled in complete online surveys (see Table 1). Most of the therapists worked in hospitals (N = 87, 68.5%). A minority could be recruited from private practices (*N* = 15, 11.8%). This classification was based on the weekly working time (hours), where more than 50% of their working time had to be allocated in the respective setting. Another 25 therapists (19.7%) reported other work settings, like universities or social institutions.

The huge majority of psychotherapists was female (104 of 127; 81.9%) and living in a relationship (84.3%). Most of them were fully licensed (54.0%), with completed studies of psychology (67.7%) or medicine (19.7%). The postgraduate therapy license of 80 (67.7%) therapists was based on CBT, of 36 (28.3%) therapists on psychodynamic therapy including psychoanalysis, of 5 (3.9%) therapists on systemic therapy, and 6 (4.7%) therapists reported combinations of the former. In the whole sample, the CBT approaches were dominant, followed by PD and Systemic Therapy.

Therapists typically start with a training in one “school,” but depending on the integrative stance of a training institute and the therapists’ curiosity, they integrate theoretical concepts and techniques of other orientations. Therefore, we cluster analyzed the therapist sample, looking for types of therapist orientations. A k-Means-Custer Analysis resulted in four well defined clusters, see Appendix 1. Two CBT and two PD Clusters were found. For both CBT and PD, there is one cluster of therapists strongly affiliated to the core of each orientation: CL2 (*n* = 34) with a strong PD orientation, almost no CBT influence and some integrative aspects (mainly Systemic, less so Humanistic, IPT or Integrative), and CL3 (*n* = 52) with a strong CBT influence and 3rd. Wave extensions. Two other clusters are also clearly primarily PD or CBT oriented, but with stronger influences from other therapies. The PD Cluster (CL1, *N* = 16) leans also into CBT, as well as a most of the other approaches (except for the most PD-like 3rd wave therapies of CBASB and Schema Therapy). An “integrative” CBT cluster (CL4, *N* = 25) is affiliated to CBT and 3^rd^ wave therapies but also with Systemic and other (humanistic /IPT and integrative) therapies.

### 3.2 Controlling for bias in case descriptions

To examine whether therapist characteristics were related to the cases’ symptoms and therapeutic challenges, we correlated both sets of variables with the therapists’ personality dimensions, attachment style, therapeutic orientation and age. We also tested for gender differences. Significance levels were adjusted using a Bonferroni correction, separately for symptoms, personality and therapeutic challenges.

Neither therapist personality variables, attachment style nor gender nor theoretical orientation were significantly correlated with any of the variables from the case descriptions. The majority of effect sizes were very small (*r*^2^ < 0.02).^1^ The maximum was *r*^2^ = 0.04, meaning that our threshold of 5% shared variance was never met (see above). Thus, we concluded that the controlled therapist variables were not biasing their case descriptions.

The workplace (inpatient unit or day hospital/private practice/other) has only a few and surprisingly moderate effects on the possible severity criteria. Patients in wards or day hospitals have a significantly worse weight situation. Wards/day hospitals have more cases with life-threatening weight (*r*^2^ = 0.049) and the lowest lifetime BMI (*r*^2^ = 0.039). In private practice, therapists see higher levels of OCD symptoms (*r*^2^ = 0.049).

### 3.3 Case description sample

The therapists reported on 127 pairs of cases - one “normal” and one “difficult” case. Despite our instruction to exclude overweight patients, we identified 12 case descriptions with BMI > 30 (max BMI under treatment). After their exclusion, our analysis sample comprises 124 “difficult” and 118 “normal” cases.

### 3.4 “Difficult” versus “normal” cases

#### 3.4.1 Clinical diagnoses

The therapists reported on 207 cases of AN including 40 with atypical AN (“difficult” cases and 98 “normal” cases), 29 cases with BN including one with atypical BN (13 “difficult” and 17 “normal” cases) and three cases diagnosed as EDNOS (two “difficult” and three “normal” cases). In the group with restrictive AN (F50.00), 28 were described as “difficult” and 40 as “normal” cases, whereas in the group with the binge-purging type of AN (F50.01) 42 patients were characterized as “difficult” and 22 as “normal” cases. Overall, there was no statistically significant difference between diagnostic groups (Chisq = 11.1; *df* = 6; *p* < 0.09).

#### 3.4.2 Spectrum of symptoms

The spectrum of symptoms showed a clear pattern of consistently worse symptom scores in the “difficult” cases, i.e., lower BMI, more binge/purging behavior, worse somatic complications etc. (see Table 3).

The symptoms showed a clear factor structure with only a few cross-factor loadings. The extracted factors were: (SF1) psychopathological symptoms, (SF2) somatic complications, (SF3) laxative/diuretics-abuse, and (SF4) binge/purging behavior. The factors (SF4) weight pathology and (SF6) OCD/somatization were least convincing, comprising two symptoms each, with weaker factorial face validity. It is important to note that the factor analysis of symptoms did not aim to construct a stable and generalizable solution. It was meant to reveal the dimensional structure of the therapists’ perception of symptoms in this particular sample of case descriptions.

#### 3.4.3 Selection of predictors

##### 3.4.3.1 Symptom spectrum

From each factor, the variable with the highest loading and the least ceiling or floor effect was included into the predictor set to discriminate “normal” vs. “difficult” cases. For example, *diuretics abuse* showed the highest factor loading (SF2 *Abuse*), yet with very low mean and variance. Consequently, it was replaced with *laxative abuse* which occurred more frequently (see Table 3).

##### 3.4.3.2 Personality functioning and personality traits

Concerning personality functioning and personality traits, the “difficult” cases again showed more personality impairment (OPD-SQS Total Score) and higher scores for pathology on all personality traits (see Table 4).

Features of borderline personality disorder (BPD) and narcissistic PD loaded highest on the first factor (PF1), while the second factor comprised anankastic and reclusive traits. Overall impairment in personality functioning (OPD-SQS Total) was loading mainly on PF2, but also substantially on PF1. Following the DSM-5 structure of (1) level of functioning and (2) personality dimensions, we decided to add the OPD-SQS total score to the model as a measure of level of functioning.

##### 3.4.3.3 Therapeutic challenges

In the “difficult” cases the therapists reported consistently more or stronger therapeutic challenges. The challenges could be allocated on three factors: (CF1) Patients’ deceiving behavior and ambivalence characterized this factor most, while co-occurring with weight-loss under treatment and pressure from external sources (e.g., families, social agencies etc.); (CF2) increasing binge-purging behavior under treatment; and (CF3) symptoms associated with traumatization (see Table 5).

### 3.5 Discrimination of D/N cases

Table 6 shows the results of the final regression, retaining the significant predictors of each group of variables (symptom spectrum, personality, challenges).

**Table 6:** Prediction of difficult/normal cases.

**Symptom spectrum**	**Model**	**Estimate**	**SE**	**Chi** ^**2**^	***p* <**
*r*^2^ = 0.17 *df* = 4; Chi^2^ = 59.82; *p* < 0.0001	Intercept	-1.329	0.238	31.30	0.0001
	Suicidality	0.555	0.132	17.56	0.0001
	Electrolyte imbalance	-0.514	0.148	12.00	0.0005
	Somatization	3.665	0.120	9.20	0.0024
**Personality**	**Model**	**Estimate**	**SE**	**Chi** ^**2**^	***p* <**
*r*^2^ =0.23 *df* = 3; Chi^2^ = 77.45; *p* < 0.0001	Intercept	-2.4846	0.533	21.76	0.0001
	PID5BF+ Antagonism	0.4922	0.107	21.38	0.0001
	PID5BF+ Detachment	0.3704	0.153	5.87	0.0154
	OPD-SQS Total Score	0.7672	0.249	9.5	0.0021
**Challenges**	**Model**	**Estimate**	**SE**	**Chi** ^**2**^	***p* <**
*r*^2^ =0.39 *df* = 2; Chi^2^ = 132.30; *p* < 0.0001	Intercept	-5.355	0.716	55.90	0.0001
	Ambivalence	1.893	0.245	59.86	0.0001
	Trauma associated symptoms	0.547	0.165	11.04	0.0009
**Final model**	**Model**	**Estimate**	**SE**	**Chi** ^**2**^	***p* <**
*r*^2^ =0.39 *df* = 3; Chi^2^ = 144.02; *p* < 0.0001	Intercept	-5.144	0.695	54.84	0.0001
	Challenges ChF1: Ambivalence	1.689	0.236	51.28	0.0001
	Symptom Spectrum SyF2: Electrolyte Imbalance	0.584	0.188	9.63	0.0019
	Personality PF2: PIDF5BF+ Antagonism	0.356	0.124	8.26	0.0040

The severity of symptoms was only marginally predictive of the perceived severity of cases. Three symptoms (*electrolyte imbalance, suicidality and somatization*) were found to significantly predict the case evaluation with only 17% of explained variance, and 68.97% of correctly identified cases. Notably, main criteria of ED diagnoses (DSM-5, ICD11) like binges or BMI were not significantly predictive of difficulty.

Three personality scores were selected to represent overall personality functioning (*OPD-SQS Total*) and discriminating factors (PIDF5BF *detachment* and *antagonism*). All three predictors were found to significantly predict D/N with 23% of explained variance. With this model 75.6% of D/N classifications were correctly identified.

In the first model on challenges (starting with three predictors: *ambivalence, more purging, symptoms associated with trauma*), the initially selected variable *more purging* was not significantly predicting D/N. Therefore, the final model comprises only two predictors (*ambivalence* and *symptoms associated with trauma*) which significantly predicted D/N with 39% of explained variance. The discrimination resulted in 80.6% of correctly identified D/N classifications.

The challenges encountered in the therapeutic process were determining the therapists’ perception of case difficulty much more than symptoms and more than personality disturbance, see Table 6 (*r*^2^ by predictor set).

#### 3.5.1 Combined variable set

When all variables of the previous models were entered into a comprehensive model, three predictors remained significant (*ambivalence, antagonism, electrolyte imbalance*). Again, we see *ambivalence* being the strongest predictor of case difficulty, the other predictors lagging behind. The amount of explained variance is very good (*r*^2^ = 43%). 81.4% of D/N categories were correctly identified, see Table 6.

The therapeutic challenge of patients being ambivalent about giving up their ED was the strongest predictor of case difficulty. Electrolyte imbalance represents the factor SF2 which summarizes the somatic comorbidity of ED. The factor analysis showed strong covariances with organ lesions and dental damage, all symptoms of advanced and chronic EDs with destructive somatic consequences (see Table 3; SF2). The PID5BF + dimension *antagonism* comprises three facets: manipulativeness, deceitfulness and grandiosity. The personality dimension (*antagonism*) and the therapeutic challenge (*deceiving behavior*) are strongly correlated in the sample, and the correlation was stronger in the “difficult” cases (*r_N_* = 0.34; *r_D_* = 0.53). Thus, in the context of an ED the PID5BF + scale antagonism is not exclusively measuring a personality trait, but also a behavior which is a challenge in treatment. Replacing *antagonism* with *deceiving behavior* in the regression model obtained very similar results.

## 4 Discussion

This study aimed to explore the therapists’ perception of “difficult” versus “normal” cases in psychotherapeutic treatments of patients with an ED. Three groups of patient variables were distinguished: symptoms and their severity, personality and personality functioning as well as possible challenges that could occur over the course of treatment. We hypothesized that challenges during the course of treatment will outperform the other predictors in their predictive value.

The group of 127 therapists who took part in the online-survey consisted of German speaking psychotherapists from whom a majority (>80%) was female and worked in a hospital context (69%). Since it can be assumed that training in psychotherapy and experience has an influence on whether a therapist perceives treatments as difficult (Zeeck et al., 2025), we placed great importance on recording the therapeutic training and influences of various therapeutic schools in detail, identifying four different clusters. However, as hypothesized, therapist` characteristics including age, gender, therapeutic training and orientation as well as attachment style, personality traits and functioning were no predictors of the therapists’ view on factors distinguishing “normal” from “difficult” cases.

The therapists reported on more cases of AN compared to treatments of patients with BN or EDNOS. Furthermore, the binge-purge subtype of AN was prominent in the group of “difficult” cases. The predominance of cases with AN may be explained by the fact that most therapists worked in hospital settings and hospital treatment is more frequently indicated for patients with AN than for patients with BN or EDNOS (AWMF, 2018).

In line with our hypothesis the “difficult” cases had higher scores in all three groups of patient variables: they were described having more and more serious symptoms, more personality pathology, and during the process of psychotherapy, the therapist was confronted with more challenges. Evaluating the predictive power of the three variable sets for case difficulty, therapeutic challenges, notably the patient’s ambivalence, showed the best estimate of case difficulty (*r*^2^ = 0.39), followed by patient personality (*r*^2^ = 0.23) and symptoms (*r*^2^ = 0.17). This way our prediction was confirmed: Case difficulty is unfolding under treatment and manifesting itself in challenges. In particular, ambivalence seems to be a challenging issue. A high ambivalence to change will be a threat to the therapeutic alliance, mainly to goal and task aspects of it. Impaired personality functioning can be assumed to affect the interaction between therapist and patients, thus also complicating therapeutic work. And finally, strong somatic symptoms need to be addressed with high priority and may induce fear. It may be necessary to interrupt psychotherapy for somatic inpatient treatment or to shift the focus of treatment from psychotherapy to strict behavioral management of eating or medical complications. The latter may happen not too often, and therefore the relationship to case difficulty may have turned out rather low in our sample. When severe somatic complications occur, however, they could dominate, interrupt or terminate a psychotherapeutic treatment.

Patients’ ambivalence is a core theme in ED treatment (Robinson et al., 2024) and a low motivation to change related to poor treatment outcome (Vall and Wade, 2015). Therefore, the German treatment guideline suggest to address ambivalence towards change throughout the whole treatment process, especially when treating patients with AN (AWMF, 2018). The specific techniques from Motivational Interviewing (MI) have been adapted for EDs (Macdonald et al., 2012) and treatment manuals for EDs usually contain specific elements in this regard as well (Denison-Day et al., 2018). Ambivalence may deny or undermine the therapeutic contract, may oppose weight gain and emphasize “positive” aspects of the eating disorder. There may be a resistance to change because a patient denies her behavior is problematic (ego-syntonic nature of the disorder), an ambivalence because of the functional role of ED symptoms (e.g., affect regulation, feeling in control) or a strong wish for autonomy, which makes it difficult to commit to treatment (Denison-Day et al., 2018). However, ambivalence is not only a threat for treatment effectiveness. In regard to the therapist, it may be a threat to self-perceived therapeutic control and therapeutic competence. This way it may also affect the therapist’s professional self and may inflict self-doubt, self-critique, perhaps helplessness and sometimes even feelings of shame and guilt, feelings which are described in ED treatments (Thompson-Brenner et al., 2012; Zeeck et al., 2025). Overall, this may be an explanation for the fact that patients’ ambivalence is a major component of perceived case difficulty.

Impairment in personality functioning and specific personality traits which more strongly affect the therapeutic alliance and which go along with manipulative behavior or deceitfulness such as antagonism, were also characterizing the difficult patient group. The importance of personality functioning is in line with previous studies, which found that a history of trauma and associated symptoms (such as self-harm) on one side and comorbid personality disorders on the other can induce negative emotional reactions in therapists including feelings of being overwhelmed, helpless, angry or frustrated (Colli et al., 2015; Bommen et al., 2023).

Finally, physical problems associated with an ED also lead to a patient being classified as “difficult” case. They will require additional medical treatment and a close cooperation with physicians, especially when it comes to acute problems such as electrolyte imbalances. However, they seem to seem to be less influential than interactional challenges in therapists’ assessments of the difficulty experienced.

One of the limitations of the study is that it only collects the subjective views of therapists. For example, even when we documented patients’ personality traits using validated questionnaires, this remains a subjective assessment without training or reliability testing. Even the memory of a patient’s minimum BMI during treatment could be distorted by memory. It would be an interesting question for future research to explore how therapists’ “objective” and “subjective” worlds diverge and which of the two most strongly influences their actions. The interpretation of results is further limited by the fact that therapists mainly worked in a hospital setting in Germany.

### 4.1 Conclusion

Besides somatic complications as indicator of symptom severity, in particular aspects related to the therapeutic process and the therapeutic relationship such as a high ambivalence of patients concerning a change and deceiving behavior characterize the group of “difficult” cases from a therapist view. The results suggest that a therapist’s ability to tolerate ambivalence and deal with interactional challenges including the management of emerging negative emotions within themselves is of great importance in the treatment of EDs.

## Data Availability

The raw data supporting the conclusions of this article will be made available by the authors, without undue reservation.

## Footnotes

1 A full table of all correlations is available on request.
