# Supplementary material for: “Normal” vs. “difficult” cases with eating disorders: the therapists’ perspective
Source: Front Psychol. 2026 Feb 18;17:1761349. doi: 10.3389/fpsyg.2026.1761349 (PMC12956787; doi:10.3389/fpsyg.2026.1761349)
Supplement: Supplementary file 1 [file Data_Sheet_1.pdf]

Supplementary Material

Supplementary Table 1: Cluster Analysis of Therapist Theoretical Orientation

	**Cluster**			
	**Cluster 1 Integrative Pdyn**	**Cluster 2 Core Pdyn with Syst.**	**Cluster 3 Core CBT / 3rd Wave**	**Cluster 4 Integrative CBT / 3rd W.**
N	16	34	52	25
**PD/Psy.analytic Th.**	3.63 (1.54)	4.32 (1.30)	0.65 (0.93)	1.60 (1.26)
**MBT**	2.19 (1.56)	2.71 (1.45)	0.71 (1.07)	1.56 (1.36)
**TFT**	0.63 (1.15)	1.38 (1.46)	0.13 (0.34)	0.72 (1.06)
**CBT Behavioral**	3.13 (0.96)	1.15 (0.74)	4.33 (1.02)	4.08 (0.95)
**CBT Cognitive**	3.00 (0.97)	1.32 (0.81)	4.54 (0.67)	4.44 (0.71)
**CBT - 3rd-Wave-C/S**	1.56 (1.26)	0.47 (0.71)	2.92 (1.19)	3.60 (1.35)
**CBT - 3rd-Wave-Mindf./Accept./Emo**	2.63 (1.41)	0.85 (0.89)	3.17 (1.17)	3.80 (1.12)
**CBT - 3rd-Wave-DBT**	3.19 (1.05)	0.53 (0.61)	2.71 (1.58)	3.28 (1.21)
**Family/Systemic TH.**	3.25 (1.24)	2.18 (1.47)	1.37 (1.14)	3.00 (1.19)
**Humanistic Therapy**	2.19 (1.42)	1.53 (1.46)	1.52 (0.67)	3.08 (1.04)
**Interpersonal Therapy**	1.94 (1.44)	1.50 (1.44)	0.56 (0.78)	3.08 (1.26)

**Integrative Therapy**	0.75 (1.00)	1.41 (1.64)	0.92 (1.30)	3.64 (1.25)

Note: Rating scale 0 = not at all to 5 = very much; MBT = Mentalization Based Therapy; TFT = Transference Focused Therapy; CBT-3rd-Wave-C/S = CBASB or Schema therapy; Mindf./Accept./Emo. = Mindfulness or Acceptance or Emotion Focused Therapy; DBT = Dialectic Behavioral Therapy
